# Post-Traumatic Osteoarthritis Assessment in Emerging and Advanced Pre-Clinical Meniscus Repair Strategies: A Review

**DOI:** 10.3389/fbioe.2021.787330

**Published:** 2021-12-22

**Authors:** Jay Trivedi, Daniel Betensky, Salomi Desai, Chathuraka T. Jayasuriya

**Affiliations:** Department of Orthopaedics, Alpert Medical School of Brown University/Rhode Island Hospital, Providence, RI, United States

**Keywords:** meniscus, repair, replacement, PTOA, osteoarthritis

## Abstract

Surgical repair of meniscus injury is intended to help alleviate pain, prevent further exacerbation of the injury, restore normal knee function, and inhibit the accelerated development of post-traumatic osteoarthritis (PTOA). Meniscus injuries that are treated poorly or left untreated are reported to significantly increase the risk of PTOA in patients. Current surgical approaches for the treatment of meniscus injuries do not eliminate the risk of accelerated PTOA development. Through recent efforts by scientists to develop innovative and more effective meniscus repair strategies, the use of biologics, allografts, and scaffolds have come into the forefront in pre-clinical investigations. However, gauging the extent to which these (and other) approaches inhibit the development of PTOA in the knee joint is often overlooked, yet an important consideration for determining the overall efficacy of potential treatments. In this review, we catalog recent advancements in pre-clinical therapies for meniscus injuries and discuss the assessment methodologies that are used for gauging the success of these treatments based on their effect on PTOA severity. Methodologies include histopathological evaluation of cartilage, radiographic evaluation of the knee, analysis of knee function, and quantification of OA predictive biomarkers. Lastly, we analyze the prevalence of these methodologies using a systemic PubMed^®^ search for original scientific journal articles published in the last 3-years. We indexed 37 meniscus repair/replacement studies conducted in live animal models. Overall, our findings show that approximately 75% of these studies have performed at least one assessment for PTOA following meniscus injury repair. Out of this, 84% studies have reported an improvement in PTOA resulting from treatment.

## Introduction

The meniscus in the knee joint is a fibrocartilaginous tissue positioned between the femoral condyle and the tibial plateau. The meniscus performs vital functions of shock absorption and provides mechanical stability to the knee joint. Unlike articular cartilage, which lines the tibial plateau and femoral condyle of the knee joint, the menisci are soft tissues that are kept in place by ligamentous attachments, and hence they can slide and contort during knee flexion/extension. The medial and lateral menisci are key structures in the knee joint that help to dissipate forces, provide stability, and lubricate and protect the articular cartilage from injury (McDermott and Amis, 2006). With increases in physically active and aged populations, incidents of meniscus injury have spiked at a significant rate. For instance, in the United States alone, meniscus injuries are encountered by 6–8% of active young adults annually and this number is increased in the elderly population (Gee et al., 2020; Kennedy et al., 2020). Initially, the meniscus was considered to be vestigial tissue, which can simply be removed surgically upon major injury, as a means of alleviating pain and restoring function to the knee joint (Chivers and Howitt, 2009; McNulty and Guilak, 2015). However, it is now known that the absence of, or injury to, meniscal tissue significantly increases the risk of post-traumatic osteoarthritis (PTOA) (Badlani et al., 2013; Chang and Brophy, 2020; Rai et al., 2020; Adams et al., 2021; Bedrin et al., 2021). Indeed, according to a recent 2020 epidemiological study of young adults, approximately 13% of patients with meniscus injuries develop OA by 18 years post-injury, whereas only about 3% of patients with uninjured knees developed the disease (Snoeker et al., 2020). While there is a strong emphasis on improving meniscus healing in emerging biomedical research, it is equally important to assess how new strategies for treating these injuries affect the development of OA in the knee. Hence, the efficacy of treatment should not rely solely on the assessment of the extent of meniscus repair, but it should also include a comprehensive evaluation of secondary/indirect outcome measures of therapeutic efficacy, such as its ability to preserve cartilage health, prevent the expression of established PTOA serum and blood biomarkers, and restore knee joint mechanics/function as closely as possible to the pre-injury state. In this review, we have cataloged recent pre-clinical therapeutic strategies that are being developed to promote meniscus healing. We further discuss the methodologies used in these studies to evaluate their respective outcome measures and detail their findings.

## Basic Meniscus Structure and Anatomy

Meniscus is divided into three zones from the outer peripheral region to the inner central thinnest region based on the degree of vascularization. The outermost region is thickest, and it is significantly vascularized. It is referred to as the red-red zone (Andrews et al., 2011; Bryceland et al., 2017). Due to the vascularization, the red-red zone is enriched in nutrients and cytokines, enabling the self-healing upon injury in this area. The inner (middle) region is referred as the red-white zone, which contains less vascularization than the red-red zone and has minimal self-healing capacity. The innermost (central) region of the meniscus is completely devoid of vasculature and lacks any ability to self-repair. This white-white zone is tapered in shape with an unattached free edge.

The lateral and medial menisci are smooth, lubricated crescent-shaped fibrocartilage tissues that partially cover and protect the articular cartilage surface from injury. In humans, the lateral and medial menisci vary greatly in their dimensions. The lateral meniscus is approximately 27–29 mm wide and 33–35 mm in length, whereas the medial meniscus ranges from 27–28 mm in width and 41–45 mm in length (Shaffer et al., 2000; McDermott et al., 2004). The lateral meniscus occupies almost one-third of the tibial plateau with the superior concave surface pivoting the femoral condyles, while the ventral surface articulates the tibial condyle (Erdemir, 2016; Zhang et al., 2020). The lateral meniscus has a constant width that occupies a large portion of the total articular surface. The medial meniscus articulates inferiorly with the medial condyle of the tibia and superiorly with the medial condyle of the femur bone. The medial meniscus occupies more than half of the contact portion of the medial compartment. The anterior horn of the medial meniscus attaches near the ACL on the tibia, whereas the posterior horn attaches above the PCL. The medial meniscus is more crescent-shaped and covers a smaller surface of the tibial plateau compared to the lateral meniscus (Makris et al., 2011; Fox et al., 2015). Reduction of meniscal tissue due to surgical resection performed as a treatment for severe meniscus tears can result in increased forces on the articular cartilage during knee loading. Veritably, the amount of meniscus tissue resected is reported to have a positive relationship with detrimental shear stress on the cartilage surfaces that can lead to OA (Vadher et al., 2006).

## Clinical Surgical Interventions for Meniscus Injuries

Meniscal injury is one of the most common sports-related knee injuries in the United States (Majewski et al., 2006; Korpershoek et al., 2017). The combined hypovascularity and hypocellularity within the white-white zone of the meniscus significantly hinders its ability to adequately heal (Athanasiou, 2009). A meniscal injury, especially one in a hard-of-healing area such as the white-white zone, becomes a strong predictor for the accelerated development of osteoarthritis (OA). This can be attributed to the undeniable importance of meniscus in maintaining knee joint health as well as the shortcomings of traditional arthroscopic meniscus repair strategies.

Deciding the best clinical course of action for surgically treating a meniscal tear not only depends on the location, nature of the injury, and tear type, but it also relies on the age of the patient and potential co-morbidities (i.e., obesity, concomitant chondral/ligamentous damage that is presented with the meniscus injury). Treatment strategies can range from minor non-surgical means such as physical therapy and the use of non-steroidal anti-inflammatory medications, to surgical means such as meniscus suturing or partial meniscectomy/resection (surgical removal of a portion of the meniscus) (Terry Canale S, 2012; Wells et al., 2021).

### Suturing and Resection

Suturing is the most conservative of the surgical approaches for meniscus repair; however, this treatment strategy has the most long-term success rate for repairing isolated tears in younger patients (Hagmeijer et al., 2019). Meniscus tears are commonly sutured according to suture placement orientation, relative to the tear: all inside (Darabos et al., 2012), outside-in (Rodeo, 2000), and inside-out (Steenbrugge et al., 2004) techniques. For more complex tears that present with tissue loss, meniscal resection is used to remove the damaged and unrepairable areas of fibrocartilage in order to reshape the meniscus to its natural semilunar shape, but this removal of tissue has been shown to alter the contact pressures within the joint (Terry Canale S, 2012). Several long-term follow-up studies in athletes and youths who have undergone either partial or complete meniscectomy suggest an increased risk of PTOA (Allen et al., 1984; Jørgensen et al., 1987; Faunø and Nielsen, 1992; Hede et al., 1992; Benedetto and Rangger, 1993; Rangger et al., 1997; Papalia et al., 2011; Longo et al., 2019).

### Meniscus Allograft Transplantation

Additionally, meniscal allograft transplantation (MAT) is implemented in cases of severe meniscal injuries which cannot be rectified using suturing or partial resection. MAT has become more frequently used as a state-of-the-art technique utilized in the pursuit of preserving/restoring the function and mechanics of the knee in severe or irreparable meniscus injuries. MAT was first described by Milachowski et al. (Milachowski et al., 1989) in 1989; since then, this strategy has been the subject of refinement in the pre-clinical and clinical settings to restore meniscus (and ultimately joint) function while preventing the development of degenerative changes in the knee (Rodeo, 2001; Lee et al., 2012). While the refined implementation of MAT has shown promising results in patients and athletes (Pereira et al., 2019; Searle et al., 2020), it still has several limitations including extrusion and shrinkage of the graft (Lee et al., 2012). The success rate for the two major scaffolds (i.e., collagen scaffolds, polyurethane scaffolds) varies from 38% to as low as 0% after 4 years of follow-up (Houck et al., 2018). The long-term results of scaffolds and allografts can be unpredictable, and it is documented that some may become non-functional due to shrinkage, extrusion, and fragmentation (Trentacosta et al., 2016; Houck et al., 2018). Taken together, these findings highlight the importance of long-term follow-up studies to inspect not only the health of the meniscus but also the entire knee in order to adequately determine the success of any treatment.

## Gauging the Success of Emerging Meniscus Repair Strategies in the Pre-Clinical Stage

Clinical assessment of long-term outcomes following meniscus repair/replacement primarily involves looking at reoperation rates for each respective surgical procedure. Other individual assessments include radiographic imaging, surveying the degree of knee pain (as reported by the patient), joint function (i.e., during walking, single hop test), and range of motion (ROM) of the knee in patients using scales such as the Knee injury and Osteoarthritis Outcome Score (KOOS) system, Lysholm, and the Tegner activity scale, to name a few. Some of these outcome measures are understandably difficult if not impractical to implement in animal models that are used in pre-clinical investigations, depending on the species that is used. For this reason, there is a set of assessments that are commonly used in pre-clinical animal studies to gain similar insight into the short and long-term efficacy of treatments.

Developing new and more effective strategies to stimulate meniscus healing is an active area of biomedical research. A number of strategies that are still in the pre-clinical stages of development focus on the use of biologics and biomimetic natural/synthetic materials, which encompass utilizing cells, bioactive factors (both chemical and biological), and injectable/implantable scaffolds, to accelerate meniscus healing (Shimomura et al., 2018). Current primary outcome measures for the success of advanced, pre-clinical meniscal repair treatments include macroscopic and microscopic (histology) assessment of meniscal fibrocartilage using routine/special/immunostaining analysis or magnetic resonance imaging (MRI) of the meniscus, and biomechanical testing of meniscal tissue. Other avenues of assessment include functional analysis of knee kinematics, quantification of secreted inflammation/catabolism associated biomarkers, and macro- and microscopic assessment of the articular surfaces and synovium for PTOA changes.

### Histological Assessment of Meniscus Repair Efficacy

Post-mortem histological assessment of meniscal fibrocartilage is currently the gold standard of evaluating the success with which a treatment stimulates healing, especially in pre-clinical animal models. Routine stains such as hematoxylin and eosin (H&E) are useful for the qualitative evaluation of newly formed tissue and its cellularity. Other stains such as Safranin O/Fast Green and Toluidine blue are used primarily to determine molecules of interest that constitute said tissue (i.e., glycosaminoglycans). Further special stains like Picrosirius red allow for the visualization of collagen matrices when imaged under polarized light, providing not only information about the collagen makeup of tissues but also enabling the detection of continuous collagen fiber bundles, which bridge newly formed tissue and pre-existing meniscus tissue at the periphery of the injury site. Furthermore, these stains allow investigators to determine how the orientation of fibers may be changed.

Immunohistochemistry involves the staining of tissue with specific antibodies to detect and visualize matrix molecules (and sometimes even soluble proteins). This technique is used to determine the presence of specific proteins or cells in a given tissue. For instance, López-Franco et al. employed COMP and Ki67 immunohistochemistry to evaluate the differences between healthy human menisci and osteoarthritic human menisci (López-Franco et al., 2016). The use of immunostaining to detect several endogenous markers such as Human Nucleus Antigen (HNA) and cell surface markers CD44 and CD99 have been employed to visualize the endogenous cell localization to the meniscal injury site (Tarafder et al., 2018). Additionally, immunostaining for CD34 and CD146 has been used to distinguish progenitor cell populations in the meniscus, which are proposed to be useful for meniscus repair applications (Osawa et al., 2013).

### Longitudinal MRI Imaging to Assess Meniscus Healing Over Time

While histology is arguably the gold standard for evaluating meniscus healing/pathology, it first requires a terminal experimental endpoint where tissue must be extracted for analysis. The benefit of the development and refinement of accurate *in vivo* imaging techniques for the assessment of meniscus healing is that it allows for longitudinal evaluation in the same subject over time. It has been established that longitudinal Magnetic Resonance Imaging (MRI) using T2 mapping is an informative means of post-injury/repair assessment (Berton et al., 2020; Yamasaki et al., 2020). Additional techniques such as T2Rho and T2* mapping are being adapted and applied to improve the sensitivity of meniscal evaluation (Koff et al., 2013; Zellner et al., 2013; Nakagawa et al., 2016). These techniques have been used to predict tissue degeneration through their ability to detect changes in fiber orientation and tissue biochemistry, including altered water, collagen, and proteoglycan content. For instance, Kondo et al. implanted synovial MSCs (syMSCs) in aged cynomolgus primates and used T1rho MRI mapping to show that this inhibited meniscal degeneration (Kondo et al., 2017). Additionally, Hatsushika et al. reported that repeated intra-articular syMSCs injections were able to stimulate healing of meniscal defects in a porcine *in vivo* model and used T2 MRI mapping to evaluate their results (Hatsushika et al., 2014). Similarly, Lucidi et al. evaluated the long-term results of medial collagen meniscal implants in human patients approximately 20 years after surgery by performing MRI scans on their knees and analyzing the results using the Yulish scoring system, which takes into account the detection of cartilage and subchondral bone elements (Lucidi et al., 2021). They found that four of the eight patients who underwent surgery developed mild OA symptoms. These studies highlight the importance of long-term longitudinal radiographic evaluation of the knee over the years in patients to evaluate the possible onset and progression of OA.

### Assessment of Meniscal Mechanical Properties Following Repair

The measurement of meniscal strength and the characterization of its mechanical compressive properties are used as a means of assessing tissue integrity. Tensile loading (to failure) is a commonly utilized method for testing tensile repair strength (Zellner et al., 2013; Nakagawa et al., 2015; Peloquin et al., 2016; Newberry et al., 2020); indentation testing is used to determine changes in tissue compression (Shen et al., 2013) (Pan et al., 2017), and pushout testing is used to evaluate the extent of shear strength due to integrative repair (Hennerbichler et al., 2007; Wilusz et al., 2008; Newberry et al., 2020). Additionally, detection of changes in the contact forces on tibial femoral articular cartilage can be used as an outcome measure of meniscal injury repair/treatment success. The most common methodologies for achieving this end include the use of MRI and CT mapping (Flanigan et al., 2010). Direct pressure mapping technologies are also commonly used to evaluate the effects of changes in knee forces following non-surgical or surgical treatment to the meniscus (Hennerbichler et al., 2007; Flanigan et al., 2010). In addition, these approaches are crucial to evaluate the effect on the cartilage surface followed by treatment when suturing or partial meniscectomy is performed.

### Assessment of Biomarkers

The quantification of protein biomarkers, as a means of detecting specific types of meniscal injuries, has more recently become an area of active investigation with the popularization of sophisticated multiplexing technologies such as Luminex, which allow nanoscale detection of robust sets of soluble protein markers. A meniscus injury is linked to the development of PTOA (Kennedy et al., 2020; Sherman et al., 2020; Tarafder et al., 2020; Adams et al., 2021). As such, the events that occur after traumatic injury or degenerative changes in the meniscus that govern the progression of OA are regulated at the molecular level. For instance, following joint injury, several inflammatory proteins (such as interleukins, TNF-alpha, Prostaglandin E2), collagenases/aggrecanases (such as matrix metalloproteinases, ADAMTS family members), and reactive oxygen species are produced (Roberts et al., 2015; Lepetsos and Papavassiliou, 2016; Roller et al., 2016; Liu et al., 2017; Clair et al., 2019). Collagenases are involved in the metabolism of cartilage and subchondral bones, and they are considered candidate biomarkers for pathological tissue remodeling upon meniscus injury or osteoarthritis (Karsdal et al., 2011; Karsdal et al., 2013). Clair et al., observed a significant upregulation of IL-6, MCP-1, MIP-1β, and MMP-13 in the synovial fluid of the patients with meniscal injury (Clair et al., 2019). These studies reveal that a range of biomarkers is modulated in the synovial fluid as well as in the serum upon injury to the meniscus. A recent study by Brophy et al., has also demonstrated that gene expression analysis of meniscal tissue obtained during arthroscopic meniscal debridement procedures may be used to predict degenerative changes in the cartilage (Brophy et al., 2017). This study reported a differential expression of the adipokines, adiponectin and resistin in meniscal tissue from knees with degenerative changes, suggesting a potential causal relationship. This study highlights the importance of biological signaling in the meniscus for maintaining articular cartilage health.

Overall, the quantification of PTOA biomarkers in the blood and synovial fluid provide a fundamental advantage over more invasive approaches, such as histopathology analysis, while also providing an additional advantage of enabling frequent analysis at multiple time points in the same subject. Analyzing synovial fluid (SF) biomarker profiles can also have advantages since SF is drawn directly from the injury micro-environment. Additionally, due to the small volume of synovial fluid, biomarkers are less diluted as compared to those dispersed into the peripheral blood.

### Functional Assessment of the Knee

Lastly, the implication on meniscus repair/replacement on longitudinal knee function is used to gauge overall efficacy in pre-clinical animal models, including both small animals such as rodents and guinea pigs and large animal models like pigs, dogs, and horses. Accurate assessment of knee function is also crucial to make informed treatment decisions, as well as to monitor debilitating effects associated with traumatic joint injury (Ericsson et al., 2006; Bremander et al., 2007; Katz et al., 2013). Gait analysis is one of most commonly used methods to evaluate knee function in the patients with meniscus injury (Bulgheroni et al., 2007; Hyodo et al., 2020; Liu et al., 2020). Similarly, unilateral and bilateral gait analysis is a mainstay in pre-clinical studies investigating the functional implication of meniscus/ACL injury and repair (Jacobs et al., 2014; Karamchedu et al., 2021). Asymmetric gait patterns are typically indicative of potential functional impairment due to the change in the normal range of motion.

### Assessment of Knee Histopathology for Signs of OA

Pre-clinical meniscus repair/replacement studies have used knee histopathology as a secondary outcome measure of success (Table 1). However, the effect of meniscus repair/replacement on the pathology of the entire knee is a crucial metric and should arguably be considered a primary outcome measure when assessing the success of treatments. Assessment of the degree of articular cartilage degradation, synovial hyperplasia, mast cell infiltration, and changes in subchondral bone are all features that can be qualitatively and/or semi-quantitatively detected by histopathology analysis of tissue sections using the same staining techniques described previously (i.e., routine/special/immunostaining) for assessing meniscus histopathology.

**TABLE 1:** Summary of our literature analysis on recent pre-clinical live animal studies.

Treatment	Animal model	Major techniques used to evaluate tissue outcomes							Meniscus (primary) outcomes	Knee health/function (secondary) outcome	Ref.
		Meniscus examination	Cartilage examination	Synovium	Sub-chondral bone	Knee function	Biomarker analysis	Radiographic evaluation			
Cells	Mouse	Histology (Saf-O), Immunofluorescence (IF)	Histology (Saf-O), Von Frey assay for OA, Mankin Score	NA	NA	NA	Ihh, Dhh, Ptch1, Gli1, Gli2, Gli3, Hhip, Smo	microCT	Collagen fibers mediated increased bridging of broken meniscus	Reduced cartilage degradation and delayed OA progression	Wei et al. (2021)
Cells	Rabbit	Macroscopic evaluation, Histology (Toluidine Blue), Cell tracking by Dil labelling	NA	NA	NA	NA	NA	MRI	Larger area and transverse diameter of regenerated tissue; greater histological scores in ADSC group than controls	NA	Takata et al. (2020)
Cells	Pigs	Histology (Saf-O, Picrosirius Red, and H&E), Immunohistochemistry (IHC)	NA	NA	NA	NA	NA	MRI	Increased Proteoglycan content; improved histological score, T2 values comparable with control menisci	NA	Ozeki et al. (2021)
Cells	Mouse	Histology (Saf-O), IHC	Histology (Saf-O), IHC	NA	NA	NA	NA	NA	Reduced inflammation; increase chondrogenesis in MSCs; increased proteoglycan content	Reduced cartilage degradation; reduced OARSI scoring	Ding, (2021)
Cells	Rabbit	Macroscopic evaluation, Histology (DMMB stain), IHC	NA	NA	NA	NA	NA	NA	Significantly enhanced regeneration of the meniscus in time dependent manner	NA	Koch et al. (2019)
Scaffold	Rabbit	Macroscopic evaluation	Histology (H&E)	NA	NA	NA	NA	NA	Scaffolds maintained integrity with no degradation of the native tissue	No degradation of cartilage surface as compared to other controls	Fan et al. (2021)
Scaffold	Sheep	Macroscopic observation, Histology (H&E) and Biomechanical	Macroscopic observation using India ink stain, Histology (Saf-O) and Biomechanical, Semi-quantitative analysis (Mankin), Indentation testing	Histology (H&E)	NA	NA	NA	NA	Most implants at the defects resembled native tissue without any signs of inflammation; increased equilibrium modules of scaffolds after 6 months	Visible softening and fibrillation of articular cartilage	Stein et al. (2019)
Scaffold	Mini Pig	Histology (Saf-O), Pauli Score, Ishida Score, MRI	Histology (Saf-O), Mankin Score	NA	NA	NA	NA	MRI	Ultimate tensile stress was similar to native tissue and scaffolds were covered with native tissue; increased cell infiltration	No cartilage degradation, fluid accumulation or Inflammation	Otsuki et al. (2019)
Scaffold	Rabbit	Macroscopic evaluation, Histology (H&E, Saf-O, Fast Green)	NA	NA	NA	NA	NA	NA	Wounds healed completely, at a faster rate and formed native tissue structure in the treatment group as compared to other controls	NA	Liu et al. (2019)
Scaffold	Sheep	Macroscopic evaluation, Histology (Saf-O, Alcian Blue, IHC)	NA	NA	NA	NA	NA	NA	Meniscal tissue repair 6-months post-implant	NA	Cojocaru et al. (2020)
Scaffold	Rabbit	Histology (H&E, Saf-O), IHC	Histology (H&E)	NA	Histology (H&E)	NA	NA	NA	Increased tissue regenerationand enhanced tissue quality	No degeneration observed on cartilage or subchondral bone	Kim et al. (2021)
Scaffold	Rabbit	Histology (H&E, Masson trichrome), IHC	Macroscopic observation using India Ink, Histology (H&E, Saf-O, Masson’s Trichrome, Alcian Blue), Modified Mankin score	NA	NA	NA	NA	NA	Enhanced cytocompatibility of the collagen coated scaffold; increased cell infiltration and ECM deposition; higher expression of COL1 in composite sponge and no significant changes in COL 2 and aggrecan	Significantly reduced cartilage degradation and considerably better gross scores and Mankin scores	Yan et al. (2019)
Scaffold	Rabbit	Histology (H&E, Sirus Red, Alcian Blue), MRI	Macroscopic evaluation, OARSI scoring, MRI	NA	NA	NA	Type I collagen and Type II collagen, Aggrecan	MRI	Increased inflammatory signals in total meniscectomy as compared to scaffold group; meniscus like tissue formation with better tensile strength	Significantly slower progression of cartilage degradation and better OARSI scoring however the scaffold group also degenerated progressively with time.	Li et al. (2020a)
Scaffold	Sheep	Histology (H&E), Macroscopic observation, IF	Histology (Saf-O, Fast Green)	NA	NA	NA	NA	NA	Scaffold retained original thickness; no anatomic variation between scaffold and native tissue, vascularization in scaffold, with increasing collagen content	No articular cartilage surface degradation	Ghodbane et al. (2019)
Scaffold	Rabbit	Histology (H&E), (H&E, Toluidine Blue), IHC, Ishida score	Histology (H&E)	NA	NA	NA	NA	X-Ray (K-L grading), MRI (WORMS assessment)	Meniscal regeneration with similar histological, biochemical and biomechanical properties as compared to native tissue	No articular cartilage surface degradation	Chen et al. (2019)
Scaffold	Rabbit	Histology (H&E), Rodeo scoring, Hayes scores, Indentation test	Macroscopic evaluation using India Ink, Indentation testing	NA	NA	NA	NA	NA	No inflammation or infection; significantly higher surface area, higher mechanical properties and higher Hayes score in scaffold group as compared to other controls	No significant difference between groups	Demirkıran et al. (2019)
Scaffold	Rabbit	Histology (H&E, Masson staining)	Macroscopic evaluation using India Ink, Histology (H&E), Mankin Score	NA	NA	NA	Col 1, Aggrecan	X-ray	Sustained release of TGF-B1 over 1-week with increased cell infiltration	Reduced cartilage surface degradation at 3 months	Wu et al. (2019)
Scaffold	Rat	Histology (H&E, Sirius Red, Saf-O), IHC (MMP-13)	Histology (H&E, Sirius Red, Saf-O), IHC (MMP-13), OARSI scoring	NA	NA	NA	IL-6, IL-8, and MMP-3	NA	Significantly reduced expression of inflammatory cytokines	Significant reduction in the cartilage surface degradation and reduced OARSI scoring	Li et al. (2021)
Scaffold	Rabbit	Macroscopic analysis and Histology (H&E, Masson’s trichrome), Quantitative modified meniscus scoring.	NA	NA	NA	NA	NA	NA	Significant regeneration of damaged meniscal tissue	NA	Abpeikar et al. (2021)
Scaffold	Sheep	Gross inspection, Histology (H&E, Saf-O) and MRI	Gross inspection, Histology (H&E, Saf-O), OARSI scoring and MRI	OARSI histopathologic synovial scoring	NA	NA	NA	MRI	No significant difference in the groups in terms of meniscal regeneration	All groups exhibited cartilage degradation, signs of synovitis, meniscal extrusion was observed in most animals	Nakagawa et al. (2019)
Scaffold	Rabbit	Histology (Toluidine Blue, Picrosirius Red, and H&E)	Histology (H&E, Saf-O), Mankin score, ICRS cartilage lesion score	NA	NA	NA	ACAN, R\FRZB, Sox9, HAPLN1, CHA Type I collagen, Type II collagen	NA	Recapitulation of healthy meniscal tissue	Reduced cartilage degradation	Sun et al. (2021)
Scaffold	Rat, Rabbit and Sheep	Histology (Toluidine Blue, Saf-O)	Histology (Toluidine Blue, Saf-O), IHC, Mankin score	NA	NA	NA	NA	microCT, X-ray	Regeneration of meniscal tissue	Improved Mankin score, Reduced cartilage degradation	Guo et al. (2021)
Scaffold	Rabbit	Histology (H&E, Saf-O, Picrosirius red), Macroscopic evaluation, IHC	Histology (H&E), OARSI scoring, Macroscopic evaluation	Histology (H&E), semi-quantitative synovitis scoring	NA	NA	Type I collagen, Type II collagen	NA	Increased reparative tissue in defects	No degradation on cartilage surface and subchondral bone	Okuno et al. (2021)
Scaffold	Rabbit	Histology (H&E, Toluidine Blue, Saf-O, Picrosirius red), IHC	Histology (Toluidine Blue, Saf-O) ICRS cartilage lesion classification Scanning Electron Microscopy	ELISA for IL-1 and TNF-alpha	NA	NA	IL-1 and TNF-alpha	NA	Meniscus structural and biomechanical properties of the scaffold group was comparable to that of native meniscus group	No degradation of cartilage surface	Li et al. (2020b)
Cell based scaffold	Rabbit	Histology (Saf-O), Ishida scoring system, IHC	NA	NA	NA	NA	NA	NA	Defect filling with fibrocartilaginous tissue	NA	Chen et al. (2020)
Cell based scaffold	Dog	Histology (H&E, Toluidine Blue, Picrosirius red), IHC	Histology (H&E, Saf-O, Fast Green), IHC	NA	NA	Lysholm score	NA	NA	Hormone (PTH) + Cells + Scaffold formed more neo-cartilage tissue compared to the cells + scaffold group with more ECM deposition	Less degenerative changes in the cartilage in treatment group	Zhao et al. (2020)
Cell based scaffold	Rat	Histology (H&E, Saf-O Toluidine Blue), IHC	microCT	NA	microCT	NA	NA	microCT	Regeneration of the meniscal tissue, reduced osteophyte formation, reduction of joint spance narrowing	Reduced cartilage degradation and delayed OA progression	Zhong et al. (2020)
Cell based scaffold	Rabbit	Histology (H&E, Saf-O)	Histology (H&E, Saf-O), IHC	NA	NA	NA	NA	NA	Prevention of meniscal extrusion; filling of meniscal defects	No cartilage degradation	Shimomura et al. (2019)
Allograft	Dog	Histology (H&E, Toluidine Blue)	Histology (H&E, Toluidine Blue)	Histology (H&E)	Histology (H&E, Toluidine Blue)	Comfortable knee range of motion (CROM), clinical lameness and limb kinetics, knee pain, knee effusion, visual analogue scale	NA	MRI	Pain, CROM, histological scores and cell viability were superior in fresh menisco-tibial group as compared to other techniques	restoring joint healt Fresh, MOPS preserved and meniscal osteochondral allograft are safe and effective to restore knee joint function	Schreiner et al. (2021)
Allograft	Sheep	Histology (H&E, Saf-O, Masson’s Trichrome, Fast Green), IHC, Ishida score, Rodeo score	Histology (Saf-O), Histopathology scoreing	NA	NA	NA	NA	NA	Three early euthanized animals showed moderate Rodeo score and low Ishida score; full-term animals showed improved Rodeo scoring, improved Ishida scoring and Improved healing	Three animals were euthanized early due to resulting knee health. Three animals (euthanized 90 days post-surgery) showed less cartilage degradation and improved histopathology scoring	Strauss et al. (2019)
Circular unidentified RNA (PDE4B)	Mouse	Histology (Saf-O)	Histology (Saf-O),OARSI Scoring	NA	microCT	Hot plate test, knee extension test and electric shock stimulated treadmill test	Aggrecan, COL2A1, SOX9, MMP13, MMP3, ADAMTS4	microCT	Increased proteoglycan content	Reduced cartilage degradation, reduced proteoglycan content, reduced OARSI scoring, and reduced discomfort and pain	Shen et al. (2021)
Extracellular vesicles	Mouse	Histology (Saf-O, Fast Green)	Histology (Saf-O),OARSI Scoring	NA	NA	NA	Aggrecan, COL2A1, ADAMTS5	NA	Increased proteoglycan content; increased proliferation and migration of chondrocyte	Reduced cartilage degradation and reduced OARSI scoring	Duan et al. (2021)
Erythropoietin	Mouse	Histology (Alcian Blue, Orange G)	Histology (Alcian Blue, Orange G)	NA	NA	NA	Type I collagen, Type II collagen, MMP13		Increased regeneration of damaged meniscal tissue; increased production of ECM; increased cell proliferation and reduced MMP-13 expression	Reduced MMP-13 expression and reduced OA severity	Fu et al. (2020)
N/A	Rabbit	Histology (H&E, Fast Green), Indentation relaxation testing	micro-CT	NA	micro-CT	NA	NA	microCT	Severe degradation in both lateral and medial menisci	Minimal changes in bone quality and morphometry, Increased osteophyte formation	Narez et al. (2021)
SDF-1	Rat	Histology (H&E, Toluidine Blue, Saf-O)	NA	NA	NA	NA	NA	NA	Enhanced migration of macrophages at the injury site, Defect repair	NA	Nishida et al. (2020)
Platelet Rich Plasma and Platelet Rich Fibrin Matrix	Rabbit	Histology (H&E)	NA	NA	NA	NA	NA	NA	No obvious benefits of PRP and PRFM on meniscus healing and matrix formation	NA	Kurnaz et al. (2020)
Local administration of Magnesium	Rabbit	Histology (H&E, Toluidine Blue), IHC	Histology (H&E, Toluidine Blue), ICRS cartilage lesion classification, Mankin score	Colony forming cells analysis	NA	NA	IL-1, TNF-alpha	NA	Enhanced adhesion, migration and fibrochondrogenesis of SMSCs in meniscus lesions	Less degradation of cartilage surface than control group, retained mechanical strength	Zhang et al. (2019)

These studies were indexed by searching PubMed for original scientific articles employing live animal models that were published between 1/1/2019 and 9/26/2021, using the following search terms in the title and abstract fields: *meniscus repair, meniscus replacement, meniscus regeneration, meniscus scaffold*. Studies are organized based on the category of therapeutic strategy tested.

## Emerging Pre-Clinical Meniscal Repair/Replacement Strategies and Their Impact on Knee Function and Pathology

Partial meniscal resection can be an effective short-term treatment for symptomatic complex meniscus tears; however, sizeable evidence shows that this approach leads to the accelerated development of OA. Similarly, MAT has its own set of challenges which were discussed above. Therefore, novel therapeutic approaches are being developed in the pre-clinical models to find effective alternative therapies. These approaches largely include the use of synthetic/biological scaffolds and/or autologous/allogeneic cells. In order to conduct a comprehensive evaluation of the efficacy of such emerging treatments, it is necessary to assess not only the repaired/replaced meniscus itself but also other important metrics that have been discussed in this article that ultimately help to assess knee functionality and/or pathology as fundamental indirect/secondary criteria. These secondary measures can help define the level of success achieved by said treatments.

In 2015, Nakagawa et al. (Nakagawa et al., 2015) performed a study in a porcine model in which they combined suture repair with allogenic MSCs enriched from synovial fluid. The study reported positive outcomes in meniscal histology, collagen deposition and tensile strength in the animal groups that received the combination treatment as compared to those that received only suture repairs (Nakagawa et al., 2015). Hatsushika et al. (Hatsushika et al., 2014), performed similar studies with larger meniscus tears and involved multiple injections of the synovium-derived MSCs. Their results supported the previous finding by noting that multiple injections is advantageous compared to a single injection (Hatsushika et al., 2014). Both studies involved acute injections immediately after the injury. Further studies in which the animals were treated after 3–4 weeks of injury demonstrated that the animal groups that are acutely treated show significantly better repair compared to those that are treated 3–4 weeks post-injury (Ruiz-Ibán et al., 2011). These studies suggest that both the chronology of the injury and the treatment is vital for meniscus injury repair. Inspection of cartilage health and/or knee function would have been most informative regarding the efficacy of this cell-based biologic treatment approach.

There are, however, many recent studies conducted in multiple animal models that have evaluated outcome measures of PTOA as important parameters of success (Table 1). For instance, Wei et al. (Wei et al., 2021) identified a novel progenitor cell population located in the horns of the meniscus that not only helps meniscus repair but also prevents cartilage degradation. The study identified Hedgehog (Hh) signaling as a crucial player in progenitor cell migration to the injury site and the inhibition of cartilage degradation. Further, they used microCT analysis to reveal that the use of Hh agonist does not induce calcification. In another study, Ding et al. (Ding, 2021) demonstrated that the intra-articular injection of BM-MSCs pretreated with c-Jun N-terminal kinase (JNK) inhibitor SP600125 in C57BL/6 mice significantly induced meniscus repair and inhibited cartilage degradation in comparison to the animals that received BM-MSCs without SP600125 pretreatment. Immunohistochemical analysis revealed that the pretreatment of SP600125 inhibits the inflammation on the articular cartilage surface suggesting SP600125 as a potential therapeutic candidate to inhibit the cartilage degradation and OA progression (Ding, 2021). Ozeki et al. (Ozeki et al., 2021) developed a meniscus injury model in micro minipigs and investigated the reparative effects of synovial MSCs on meniscus injury repair. They used cartilage surface degradation as an outcome measure. Histological evaluation and MRI analysis indicated that synovial MSCs improved meniscus healing and also protected the articular cartilage surface from degradation as compared to the controls (Ozeki et al., 2021).

**FIGURE 1:**
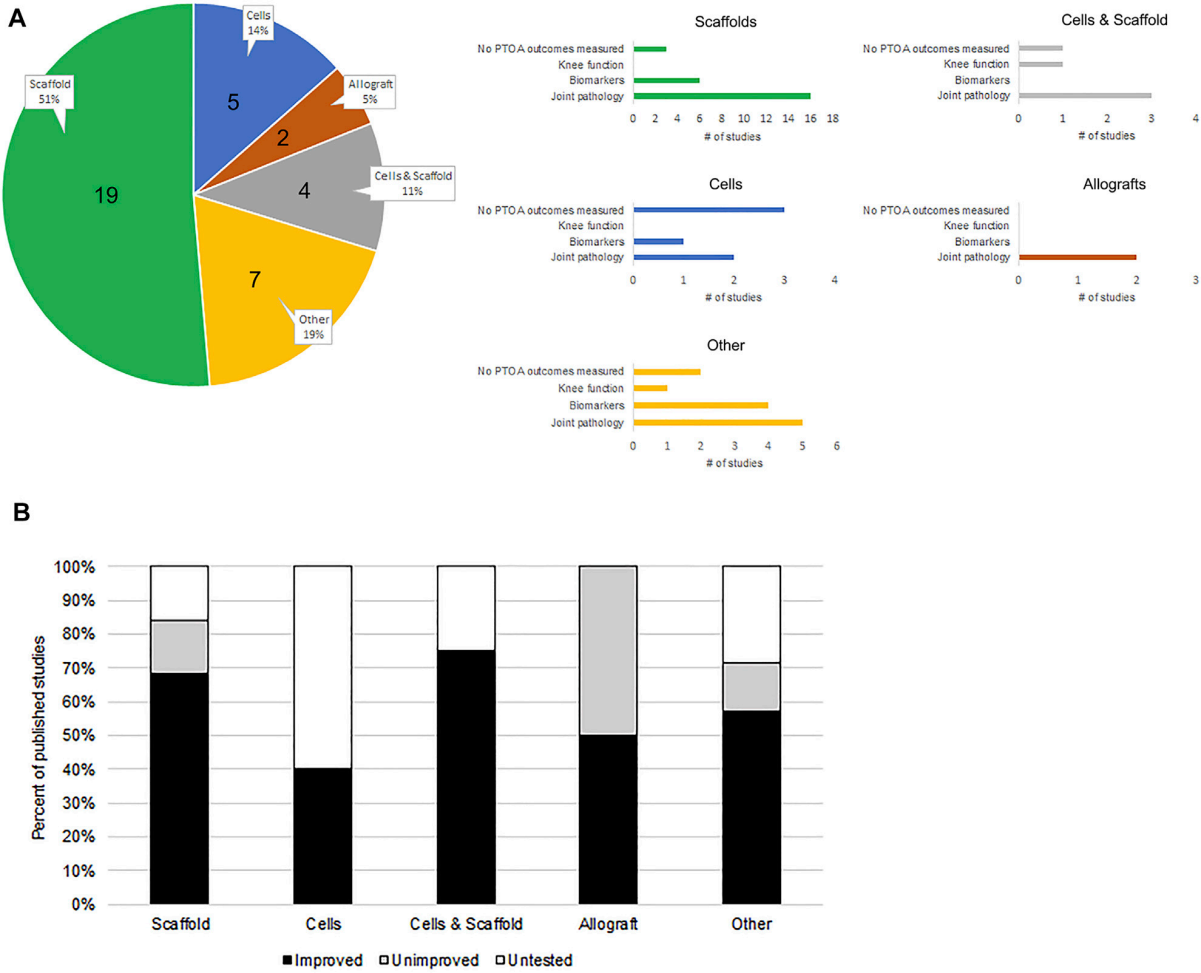
Recent pre-clinical meniscus treatment studies in animal models and their PTOA attenuation success rate. **(A)** The 37 studies indexed in Table 1 were used to extrapolate the distribution of published investigations that utilize different strategies for meniscus injury treatment. Studies have been categorized based on their use of cells, scaffolds, cell and scaffold combination, allografts, or altogether different (other) strategies, by a percentage of all indexed studies. The number of studies is indicated in the pie chart. These categories are further broken down (see respective bar graphs) based on secondary outcome measures used to evaluate therapeutic success, including knee function, mRNA/protein biomarker analysis, and joint pathology detection. Note that some studies have performed more than one type of analysis. **(B)** Therapy categories were further broken down based on percent distribution of studies in each that reported knee PTOA outcomes.

Further, in a recent study by Shimomura et al. (Shimomura et al., 2019), the investigators used an MSC monolayer sheet wrapped around an electrospun nanofiber scaffold to treat a radial meniscal injury in rabbits. In addition to evaluating the meniscus, they conducted a histological evaluation of the articular cartilage and showed that there was significantly less degradation in the cell scaffold treated animals, compared to the control animals. Zhong et al. (Zhong et al., 2020) utilized a rat model of meniscus injury-induced osteoarthritis to investigate the efficacy of an injectable extracellular matrix (ECM) hydrogel on meniscus repair and OA development. Radiographic evaluation using micro-CT scans revealed that the ECM hampers osteophyte formation, prevents narrowing of the joint space, and inhibits OA development. Table 1 is a comprehensive list of meniscus repair/replacement studies in live animal models that were published and referenced on PubMed in the past 3-years. Table 1 summarizes the outcomes and assessment techniques used to evaluate the meniscus itself, knee function, and knee health. Figure 1 illustrates the distribution of these recent studies based on the implemented therapeutic techniques and their efficacy in the assessment of PTOA outcomes.

Besides cell/scaffold-based pre-clinical investigations of meniscus injury treatment, other less common approaches have also been explored. Some studies that use these approaches also include the additional evaluation of outcome measures that do not directly address the health of the meniscus, but rather the health of the surrounding tissue. For example, Shen et al., recently identified a circulating RNA, circPDE4B, as a potential target for the prevention of cartilage degradation and OA progression upon meniscus injury in mice. Histological evaluation of the mouse knee suggested that adeno-associated virus mediated delivery of circPDE4B inhibits the cartilage matrix breakdown, which was otherwise observed in the control mice knees. Fu et al. (Fu et al., 2020) investigated the actions of Erythropoietin (EPO) in meniscus organ culture and reported the mechanistic details of its effects on meniscus regeneration. Their histological analysis using Alcian Blue and Orange G staining revealed that the EPO treatment significantly reduced the degradation of the cartilage surface and ultimately attenuated OA development. Another recent study by Duan et al. (Duan et al., 2021) underscored the crucial role of extracellular vesicles (EVs) in OA progression upon meniscus injury. Through extensive *in vitro* and *in vivo* characterization, the authors revealed that EVs secreted by lipopolysaccharide enriched synovial MSCs inhibit aggrecan and collagen 2 reduction and attenuate the upregulation of IL-1β responsive ADAMTS5. They used histological analysis to reveal that the EVs significantly prevented joint degeneration in a mouse model of OA.

## Conclusion

The detection of PTOA development in response to meniscus injury can be achieved using metrics designed to evaluate knee function, its inflammatory microenvironment (through biomarker detection), and the histopathology of tissues in the joint. Our analysis of recent scientific literature shows that 84% of studies involving the use of meniscus scaffolds and 100% of studies using allografts for meniscus injury treatment have examined at least a single outcome measure that reveals the effect on overall knee function/health to help gauge treatment success. On the other hand, only 40% of studies that use cells, and 71% of studies involving non-conventional (other) treatment approaches, have done the same. Altogether, we have found that 75% of all recent studies catalogued in our search have examined at least a single outcome assessment that is relevant to PTOA, as part of the analysis. Approximately 84% of these studies have reported an improved outcome due to treatment.
